# The relationship between glasgow prognostic score and serum tumor markers in patients with advanced non-small cell lung cancer

**DOI:** 10.1186/s12885-015-1403-x

**Published:** 2015-05-10

**Authors:** Ai-Gui Jiang, Hong-Lin Chen, Hui-Yu Lu

**Affiliations:** 1 Department of Respiratory Diseases, Jiangsu Taizhou People’s Hospital, Yingchun Road 210#, Taizhou City, 225300 Jiangsu Province P.R. China; 2 Nantong University, Qixiu Road 19#, Nantong city, 226001 Jiangsu Province P.R. China

**Keywords:** Advanced non-small cell lung cancer, Glasgow prognostic score, CYFRA21-1, CEA, TPS

## Abstract

**Background:**

Glasgow Prognostic Score (GPS) has been reported as a powerful prognostic tool for patients with advanced non–small cell lung cancer (NSCLC). The aim of this study was to assess the relationship between GPS and prognosis related tumor markers in patients with advanced NSCLC.

**Methods:**

We included 138 advanced NSCLC patients and twenty healthy controls in the study. GPS was calculated by combined serum C-reactive protein (CRP) and albumin. Three serum tumor markers, which included cytokeratin 19 fragment antigen 21-1 (CYFRA21–1), carcinoembryonic antigen (CEA) and tissue polypeptide specific antigen (TPS), were detected by enzyme-linked immunosorbent assay (ELISA). GPS and tumor markers were all assessed before chemotherapy. All patients received at least 2 courses of cisplatin-based chemotherapy. After that, 2 to 5 years follow-up was conducted.

**Results:**

Median levels of CYFRA21–1 were 1.5 ng/ml (0.1–3.1 ng/ml) in healthy controls, and 4.6 ng/ml (0.7–35.2 ng/ml) in GPS 0 advanced NSCLC, 11.2 ng/ml (0.4–89.2) ng/ml in GPS 1 advanced NSCLC, and 15.7 ng/ml (2.9–134.6 ng/ml) in GPS 2 advanced NSCLC, respectively. Median levels of CYFRA21-1 were higher in NSCLC patients than in healthy controls, and CYFRA21-1 increased gradually according to GPS category in NSCLC patients (*P* < 0.05). Similar results were found for median levels of CEA and TPS in healthy controls and NSCLC patients (*P* < 0.05). In NSCLC patients, positive correlations were found between CYFRA21-1 and GPS, CEA and GPS, TPS and GPS. The Spearman’s rank correlation coefficient were 0.67 (*P* < 0.05), 0.61 (*P* < 0.05) and 0.55 (*P* < 0.05), respectively. Survival analyses showed GPS was an independent prognostic factor for advanced NSCLC. CYFRA21-1(>3.3 ng/ml) and TPS (>80 U/l) were related with the prognosis of advanced NSCLC by univariate analyses, but multivariate analyses showed CYFRA21-1, TPS and CEA were not the independent prognostic factors for advanced NSCLC.

**Conclusions:**

Our results showed GPS were positive correlated with CYFRA21-1, CEA and TPS in patients with advanced NSCLC. However, GPS was more efficient in predicting prognosis of advanced NSCLC than these three single prognosis related tumor markers.

## Background

Although many progresses have been made in targeted therapy, chemotherapy and radiotherapy in recent years, the prognosis of advanced Non-small cell lung cancer (NSCLC) is still poor, with the median overall survival 30.5 months and the median progression-free survival 10.8 months [1]. Accurate prediction of prognosis outcome in advanced NSCLC also remains challenging. Even within the same stage, same performance status, same treatment group and same response to treatment, survival varies from patient to patient [2].

Inflammatory responses play decisive roles at different stages of tumor development, including initiation, promotion, malignant conversion, invasion and metastasis [3]. Glasgow prognostic score (GPS), a inflammation-based scoring system, was found an useful tool in predicting prognosis for gastric cancer [4], colorectal cancer [5], pancreatic cancer [6], hepatocellular cancer [7], esophageal cancer [8], and cervical cancer [9]. In NSCLC patients, our previous study has also found GPS was a useful and important predictor of progression free survival (PFS) and overall survival (OS) [10]. This conclusion also confirmed by other studies [11–13].

In addition, several tumor markers have been described to be independently relevant for estimation of prognosis in terms of overall or progression-free survival in NSCLC patients [14]. Cytokeratin 19 fragment antigen 21–1 (CYFRA21-1) is a cytokeratin expressed in simple epithelium, which has been extensively studied in patients with NSCLC has been demonstrated to be clinically useful. Serum concentrations of CYFRA 21–1 correlate with tumor burden, and CYFRA 21–1 is an independent prognostic factor for NSCLC [15–17]. Carcinoembryonic antigen (CEA) is an oncofetal glycoprotein of the cell surfaces. The NSCLC patients with a persistently high serum CEA level after had worst prognosis [18, 19]. One study showed patients with normal preoperative serum CEA levels had better 5 year survival than patients with high preoperative serum CEA levels (71.1 % versus 54.6 %, *P* = 0.016) [20]. Tissue polypeptide specific antigen (TPS) is another important prognosis related tumor markers which has been confirmed by many studies [21, 22].

GPS and some tumor markers also have prognosis predicting value in patients with NSCLC. However, the relationship between GPS and tumor markers level is still unknown. The aim of the present study was to examine the relationship between an inflammation-based GPS and prognosis related tumor markers (CYFRA21-1, CEA and TPS) level in patients with NSCLC.

## Methods

### Patients

Between January 2008 and January 2011, consecutive patients with stage IIIB or IV NSCLC were enrolled in this prospective cohort study. All NSCLC diagnosis was confirmed by cytological or histological examination. Clinical staging was based on clinical findings, chest X-ray, computed tomography of the chest, abdomen, brain and bone scintigraphy. Basic demographics, which included age, gender, histological type, smoking status and Eastern Cooperative Oncology Group Performance Status (ECOG’s PS) were recorded before chemotherapy. We also enrolled 20 healthy volunteers as control group. The volunteers have compared age, sex and smoking status with NSCLC patients. The study was approved by the Ethics Committee of Taizhou hospital, and all patients and healthy control signed an informed consent before inclusion in the study.

### GPS system

GPS were defined by combined serum C-reactive protein (CRP) and albumin [4–13]. Patients with a CRP <10 mg/L and albumin >35 g/L were allocated to GPS 0. If only CRP was increased or albumin decreased patients were allocated to the GPS 1, and patients in whom CRP was >10 mg/L and albumin level <35 g/L were classified as GPS 2.

Before chemotherapy, 10 ml blood sample was collect. 5 ml sample was sent to the laboratory immediately. CPR and albumin concentration were examined by routine laboratory measurements. After that, GPS was calculated.

### Serum tumor markers

The remaining 5 ml serum sample was stored at −20 °C for future analysis. CYFRA21-1, CEA and TPS measured by enzyme-linked immunosorbent assay (ELISA) using commercially available assay kits (Immuno-Biological Laboratories, Gunma, Japan). All operations were followed by manufacturer’s instructions. As recommended by the manufacturers, the following cut-offs for serum levels were used initially: CYFRA21-1 3.3 ng/ml, CEA 5 ng/ml, and TPS 80 U/l.

### Treatment and follow-up

Patients with ECOG’s PS 0–1 received at least 2 courses of cisplatin-based chemotherapy and received courses until the appearance of progressive disease. The cisplatin-based regimens were vinorelbine (25 mg/m^2^) on days 1 and 8 plus cisplatin (80 mg/m^2^) on day 1 of a 21-day cycle, and gemcitabine (1000 mg/m^2^) on days 1 and 8 plus cisplatin (80 mg/m^2^) on day 1 of a 21-day cycle. Patients with ECOG’s PS 2 received docetaxel (75 mg/m^2^) on days 1 and docetaxel (35 mg/m^2^) on days 1, days 8 and days 21 every 3 weeks. Patients with ECOG’s PS 3 only received best support care.

All patients received 3 to 5 years follow-up. The outcomes included progression free survival (PFS) and overall survival (OS).

### Statistical analysis

Data are presented as medians, with ranges. The chi-square test was used for categorical data (compare characteristics between NSCLC patients and healthy controls). The Kruscal-Wallis H test was used for non-normal distribution continuous data for more than two populations (compare tumor markers between GPS 0, GPS 1, GPS 2 NSCLC patients and healthy controls). Associations between GPS and the level of serum tumor markers were analyzed using Spearman’s rank correlation coefficient. Survival analyses were conducted by univariate Kaplan–Meier method and multivariate Cox proportional hazards model. Results were presented as hazard ratio (HR) with 95% confidence interval (95% CI). *P* < 0.05 was considered significant. All statistical analyses were performed using IBM SPSS statistics software (version 19.0, IBM, Armonk, NY).

## Results

### Characterization of NSCLC patients and healthy controls

One hundred thirty-eight NSCLC patients were included in the study. Patients’ median age was 55 years (range, 37–81years), 63 (45.7 %) patients >60 years, 117 (84.8 %) patients were male, and 42 (30.4 %) patients were smokers. 20 healthy controls and 138 NSCLC patients were similar in terms of age, gender and smoking status. In 138 NSCLC patients, 67 (48.6 %) patients had squamous cell carcinoma and 56 (59.6 %) had stage IV disease. and 82 (59.5 %) patients had an ECOG performance status 0 or 1. Characteristics of healthy controls and NSCLC patients were listed in Table 1.

**Table 1:** Characteristics of healthy controls and NSCLC patients

Characteristics	Healthy controls (n = 20)	NSCLC patients (n = 138)	*χ* ^2^	*P* Value
Age (≤60/>60)	12/8	75/60	0.140	0.709
Gender (M/F)	16/4	117/21	0.300	0.584
Smoking status (Y/N)	6/14	42/96	0.002	0.968
Histologic type (Squ/Ade/Oth)	N/A	67/48/23	N/A	N/A
Stage (IIIB/IV)	N/A	53/83	N/A	N/A
Performance status (0/1/2/3)	N/A	35/47/32/24	N/A	N/A

Squ, Squamous cell carcinoma; Ade, Adenocarcinoma; Oth, Others; N/A, Not applicable.

### Relationship between GPS and serum tumor markers

**Fig. 1:**
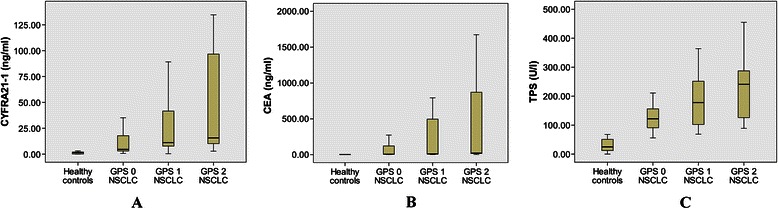
The distribution of three tumor markers in healthy control (n = 20) and NSCLC patients (GPS 0 n = 95; GPS 1 n = 32; GPS 2 n = 11). **A**: cytokeratin 19 fragment (CYFRA21-1) concentration was significant deferent between four groups (P<0.05); **B**: carcinoembryonic antigen (CEA) concentration was significant deferent between four groups (P<0.05); **C**: tissue polypeptide specific antigen (TPS) concentration was significant deferent between four groups (P<0.05)

Median levels of CYFRA21-1 were 1.5 ng/ml (0.1–3.1 ng/ml) in healthy controls. In NSCLC patients, median levels of CYFRA21-1 were 4.6 ng/ml (0.7–35.2 ng/ml) in GPS 0, 11.2 ng/ml (0.4–89.2) ng/ml in GPS 1, and 15.7 ng/ml (2.9–134.6 ng/ml) in GPS 2, respectively. The Kruscal-Wallis H test showed median levels of CYFRA21-1 were significant different between four groups (*P* < 0.05). Median levels of CYFRA21-1 were higher in NSCLC patients than in healthy controls. In NSCLC patients, median levels of CYFRA21-1 increased gradually according to GPS category. Similar results were found for median levels of CEA and TPS in healthy controls and NSCLC patients (*P* < 0.05). Figure 1 showed the trends of 3 serum tumor markers in healthy controls and NSCLC patients.

In NSCLC patients, positive correlation was found between CYFRA21-1 and GPS. The Spearman’s rank correlation coefficient was 0.67 (*P* < 0.05). Positive correlations were also found between CEA and GPS, TPS and GPS. The correlation coefficient were 0.61 (*P* < 0.05) and 0.55 (*P* < 0.05), respectively.

### The relationship between advanced NSCLC prognosis and GPS and serum tumor markers

Univariate analyses showed GPS was related with the prognosis of advanced NSCLC. After adjusted by patients’ age, gender, smoking status, histologic type, tumor stage, performance status and serum tumor markers, the multivariate analyses confirmed that GPS was an independent prognostic factor for advanced NSCLC.

For serum tumor markers, CYFRA21-1(>3.3 ng/ml) and TPS (>80 U/l) were related with the prognosis of advanced NSCLC by univariate analyses, but multivariate analyses showed CYFRA21-1 and TPS were not the independent prognostic factors for advanced NSCLC. In univariate analyses and multivariate analyses, CEA (>5 ng/ml) also didn’t show the relationship with the prognosis of advanced NSCLC.

**Fig. 2:**
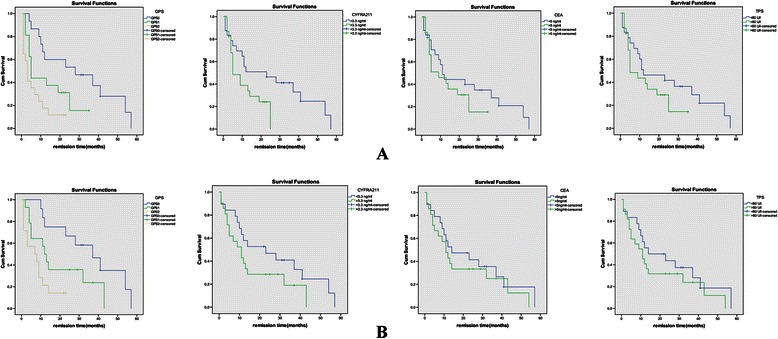
Survival curves Stratified by GPS and serum tumor markers. **A**: for the progression-free survival. **B**: for the overall survival

Details of univariate and multivariate survival analyses were listed in the Table 2, and survival curves stratified by GPS and serum tumor markers were shown in Fig. 2.

**Table 2:** Relationship between advanced NSCLC prognosis and GPS and serum tumor markers in 138 patients

Characteristics	Disease-Free survival				Overall survival			
	Univariate		Multivariate		Univariate		Multivariate	
	HR(95% CI)	*P*	HR(95% CI)	*P*	HR(95% CI)	*P*	HR(95% CI)	*P*
GPS								
0	1	0.01*	1	0.03*	1	0.02*	1	0.02*
1	0.7(0.4–0.9)		0.8(0.5–0.9)		0.8(0.5–0.9)		0.8(0.4–0.9)	
2	0.5(0.2–0.8)		0.6(0.2–0.8)		0.5(0.3–0.8)		0.5(0.2–0.9)	
CYFRA21-1 (>3.3 ng/ml)	0.6(0.5–0.9)	0.03*	0.7(0.5–1.0)	0.08	0.7(0.3–0.9)	0.04*	0.8(0.5–1.0)	0.07
CEA (>5 ng/ml)	0.7(0.4–1.1)	0.13	0.7(0.5–1.2)	0.17	0.8(0.5–1.1)	0.19	0.8(0.6–1.3)	0.19
TPS (>80 U/l)	0.7(0.5–0.9)	0.04*	0.7(0.5–1.1)	0.12	0.8(0.5–1.0)	0.05	0.8(0.4–1.1)	0.13

*P < 0.05.

## Discussion

Lung cancer is the most common cancer in world. Each year, nearly 1,708,800 patients were diagnosed with lung cancer and over 1,378,400 die, corresponding to an annual age-standardized rate of 47.4 cases per 100,000 patients, annual age-standardized mortality rate of 39.4 deaths per 100,000 in more developed areas [23]. GPS was found a useful prognosis predictor in patients with NSCLC. Serum tumor markers, such as CYFRA21-1, CEA and TPS have been also confirmed as important prognosis risk factors for NSCLC [14–22]. The aim of the present study was to examine the relationship between GPS and prognosis related tumor markers (CYFRA21-1, CEA and TPS) level in patients with NSCLC. We found the median levels of CYFRA21-1, CEA and TPS were all higher in patients with NSCLC compared with healthy controls. In patients with NSCLC, CYFRA21-1, CEA and TPS were all increased gradually according to GPS. The Spearman’s rank correlation showed positive correlations existing between these three tumor markers and GPS in NSCLC patients. Brown DJ and his colleagues also compared GPS and serum biochemical variables in patients with advanced lung and gastrointestinal cancer, they also found found GPS were correlated with the biochemical variables, which included sodium, chloride, creatine kinase, zinc, vitamin D, calcium, copper, alkaline phosphatase, gamma-glutamyl transferaseand lactate dehydrogenase [24]. This conclusion was same as our study.

Some possible mechanisms maybe explain these correlations between GPS and tumor markers. GPS is a cancer prognosis predicting system based on inflammation scoring. Studies have confirmed that inflammatory activate immune cells product cytokines, such as NF- κB, STAT3, AP-1, FOXP3 and interleukin, which can stimulate cancer cell proliferation and survival. That is a major tumor-promoting mechanism for inflammatory [3]. A recent study showed the relative expression of transcription factor FOXP3 tended to increase expression of cytokeratin 19 [25]. Kim et al. also found transcription factor NF-κB were related with elevated carcinoembryonic antigen level [26]. For tumor marker TPS, Kramer et al. found TPS increased along with interleukin-8 (IL-8) [27]. Inflammation promote angiogenesis is another important mechanism for tumor promote and metastasis. Important proangiogenic genes, such as VEGF, CXCL1, CXCL8, IL-8 and HIF1a, are directly regulated by inflammatory cytokines [3]. Yang et al. reported the level of cytokeratin 19 was related to tumor angiogenesis [28]. VEGF is regarded as the strongest angiogenic factor, which has been found related with carcinoembryonic antigen [29].

Although we found GPS are positive correlated with these tumor markers (CYFRA21-1, CEA and TPS) and in advanced NSCLC patients. Survival analyses showed GPS was an independent prognostic factor for advanced NSCLC. While CYFRA21-1(>3.3 ng/ml), CEA (>5 ng/ml), and TPS (>80 U/l) were not the independent prognostic factors for advanced NSCLC. It seems GPS was more efficient in predicting prognosis of advanced NSCLC than these three single prognosis related tumor markers. No other studies were found for assessing prognosis of lung cancer by combine GPS and tumor markers. While in colorectal cancer patients, Choi KW et al. found CEA and GPS were associated with cancer-specific survival in univariate analysis, but only GPS was identified as independent prognostic factors in multivariate analysis [30]. In gastric cancer patients, Jiang X et al. reported increased GPS, elevated CEA and CA19-9 predicted a higher risk of postoperative mortality in both relative early-stage (stage I; *P* < 0.001) and advanced-stage cancer (stage II, III and IV; *P* < 0.001) in univariate Kaplan-Meier analysis; but in multivariate analysis, only GPS predicted postoperative mortality (OR 1.845; 95% CI 1.184–2.875; *P* = 0.007), not CEA (OR 1.234; 95% CI 0.955–1.595; *P* = 0.107) and CA19-9 (OR 1.213; 95% CI 0.916–1.605; *P* = 0.177) [31]. These results were same like our study.

There was a limitation in our study. We only investigated the relationship between GPS and tumor markers before chemotherapy. Chemotherapy treatment may change GPS and serum tumor markers level, and the change of GPS and serum tumor markers level may be related with chemotherapy response. We don’t know whether this relationship will continue to exist. However, some blood samples were lost in the follow-up.

## Conclusions

The results of the present study show that GPS were positive correlated with CYFRA21-1, CEA and TPS in patients with advanced NSCLC. However, GPS was more efficient in predicting prognosis of advanced NSCLC than these three single prognosis related tumor markers.
